# Ternary composite amendments of sludge-humic acid-fertilizer for mineral mud paddy field utilization

**DOI:** 10.1016/j.isci.2026.116551

**Published:** 2026-07-03

**Authors:** Zongwei Zhu, Zhihao Wang, Yusong Kong, Jing Liang, Gongning Chen, Lihao Zhang

**Affiliations:** 1 Guilin Sustainable Development Promotion Center, Guilin 541199, China; 2 Guangxi Key Laboratory of Environmental Pollution Control Theory and Technology, Guilin University of Technology, Guilin 541006, China; 3 University Engineering Research Center of Watershed Protection and Green Development, Guilin University of Technology, Guilin, Guangxi 541006, China

**Keywords:** mineral mud, ternary composite amendment, paddy field utilization, ecological restoration

## Abstract

To address severe challenges posed by the contamination of mineral mud, a ternary composite comprising sludge, humic acid, and silica-calcium-magnesium (Si-Ca-Mg) fertilizer was developed. The system aims to achieve multi-dimensional paddy field utilization through synergistic mechanisms with mining mud. Field experiment results demonstrated that the amended mineral mud exhibited a pH increase of 9.46%–13.07% and significant improvements in nutrition, thus showing higher rice yields compared with the control group. The amendments promoted soil particle aggregation; consequently, heavy metals in the mineral mud tended to be *in-situ* deposited, rather than being taken up by plants, which also confirmed the reduction of the health risk assessment. Microbial community analysis revealed that the composite amendments significantly influenced the diversity of sludge microbial communities. The findings provide a theoretical foundation for the remediation of mineral mud and the ecological restoration of mining areas.

## Highlights

• Achieved the mineral mud paddy field utilization
• The composite conditioner reduced soil risk indices
• The yields increased over 200 times compared with the original
• The physicochemical properties of the soil were analyzed

## Graphical abstract

**Figure:**
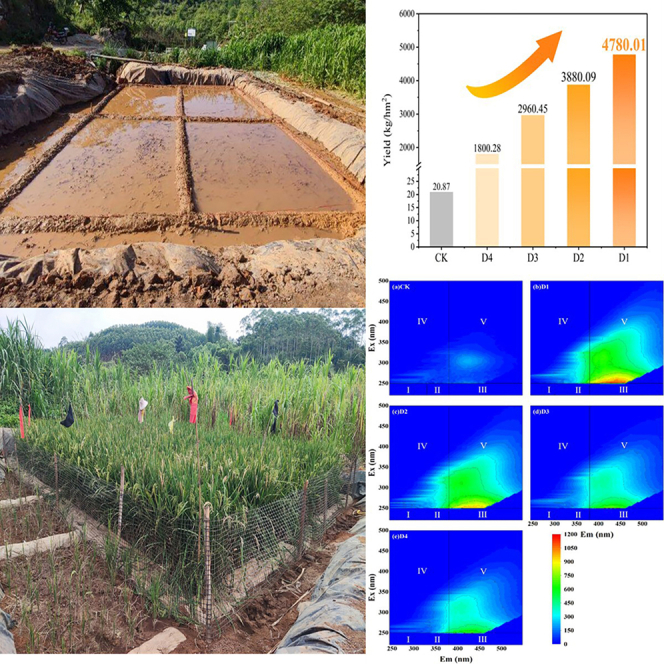

## Teaser

Soil science; agricultural science

## Introduction

Mineral mud, a typical solid waste generated from mining activities, has become a global environmental challenge due to its massive accumulation. In China, the stockpiled mineral mud currently exceeds 350 million tons, while Western countries have accumulated over 8 billion tons.^1^ The mineral mud contains substantial amounts of heavy metals (e.g., Cd, Pb, and As), sulfides, and acidic compounds. Long-term, open-air stockpiling or improper landfilling of mineral mud leads heavy metal leaching and surface runoff, posing severe threats to adjacent paddy ecosystems.^2^ In contaminated paddy soils, heavy metal concentrations can be several times higher than background levels, causing soil compaction, acidification, and suppressed microbial activity.^3^ More critically, uptake of these metals by plants introduces significant risks to food security and human health through bioaccumulation in the food chain.^4^

Current technologies for mineral mud treatment can be classified into three main categories: physical containment, chemical stabilization, and bioremediation. Physical containment methods, while effective in rapidly preventing pollutant dispersion, suffer from high operational costs and inefficient land use.^5^ Chemical stabilization techniques typically involve the addition of lime, phosphates, or other immobilizing agents to reduce heavy metal mobility, but the chemical agents may inadvertently cause secondary issues such as soil salinization or nutrient imbalance.^6^ Bioremediation approaches, despite environmental friendliness, are limited by lengthy remediation periods and poor adaptability to highly acidic soils.^7^ Resource utilization needs to address more complex applications.^8^^,^^9^ Achieving soil structure reconstruction and functional restoration through synergistic interactions among multiple components presents a new avenue for the reuse of mineral mud. By combination, the effects and characteristics of individual amendments are organically integrated. For example, humic acid (HA) enhances soil structure, fertility, and pH regulation, promoting soil aggregate formation, nutrient release, and microbial community optimization.^10^ The carboxyl and phenolic hydroxyl groups in HA molecules carry negative charges. They can have ion exchange and complexation reactions with heavy metal cations, affecting soil adsorption and migration of heavy metal ions.^11^ Tests show that HA increases the cation exchange capacity (CEC) of soil by 58%, indicating a significant promoting effect of humus addition.^12^ Silica-calcium-magnesium (Si-Ca-Mg) fertilizer can increase the pH of acidified soil, reduce the content of exchangeable heavy metals like cadmium, lower their availability, and reduce rice cadmium uptake.^13^ A reduction in available soil cadmium by 39.08%–46.40% also a significant yield-increasing effect, and an average increase of 18.8%–27.6% was observed by Min et al.^14^ Mineral mud accumulation is often accompanied by structural degradation, nutrient deficiency, and biotoxicity. These simultaneous problems can be addressed through organic–inorganic composite multifunctional amendments by adjusting their formulation ratios to achieve synchronous restoration. This approach improves soil structure, regulates nutrient supply, reduces biotoxicity, and supports plant growth.^15^ Concurrently, soil remediation efficiency and usability necessitate consideration of soil environmental characteristics (pH, organic matter, chemical composition, etc.). Xu et al.^16^ investigated the remediation effects of an aluminum sulfate-biochar agent on semi-arid grasslands, finding that compared with individual additions, the composite system more effectively drove changes in soil physicochemical properties, manifesting as significant increases in vegetation biomass and root biomass. Furthermore, utilizing industrial wastes as matrix materials for multi-component remediation agents represents a key focus in resource-efficient soil remediation. Taking sludge as an example, wastewater treatment residual sludge is rich in organic matter and nitrogen-phosphorus nutrients. After high-temperature composting, it can serve as soil amendments.^17^ Sludge, as an amendment, can improve soil pore structure and enhance its water-holding capacity. However, when used alone, it increases copper and zinc leaching by 12%–20%, necessitating its combination with other materials.^18^

Organic-inorganic composite amendments have been widely applied for the remediation and reclamation of heavy metals in agricultural soils. In terms of improving crop yield, Hamid et al.^19^ used a combined amendment (GSA-4) to more effectively immobilize heavy metals in contaminated paddy fields, thereby ensuring the safe production of rice (with cadmium and lead concentrations in grains reduced by 77% and 88%, respectively). Keskinen et al.^20^ reported that the combined application of inorganic and organic amendments altered soil nutrient availability, thereby affecting metal bioaccumulation. Existing studies have investigated the remediation mechanisms following the application of composite amendments, including changes in soil water content, nutrient elements, physicochemical properties, and microbial succession.^21^^,^^22^^,^^23^ Although the efficacy of multi-component amendments in simple soils has been demonstrated, knowledge gaps remain regarding the remediation processes and mechanisms in extreme soil environments such as mineral mud, which is characterized by low nutrient content, high heavy metal contamination, and poor soil structure.

In general, and based on the research within the group, it was chosen to construct a ternary composite amendment system comprising sludge, HA, and Si-Ca-Mg fertilizer, and to investigate the organic-inorganic synergistic mechanism of this system. Sludge provides a source of readily available nutrients, HA enhances long-term organic chelation capacity, and Si-Ca-Mg fertilizer regulates pH and stabilizes heavy metal forms.^24^^,^^25^ Field experiments were conducted using the ternary composite amendment. By comparing the effects of different ratios of the composite amendment on the physicochemical properties, crop growth, and microbial activity of mine tailings in practical applications, we evaluated its enhancement effect on the ecological functions of mine tailings and analyzed the mechanisms underlying its paddy field conversion improvement. The research findings will provide theoretical foundations and technical support for the paddy field conversion of mineral mud. The combined use of these three components holds promise in addressing the limited effectiveness of single amendments, enhancing scientific backing for the engineering application of mineral mud paddy field conversion.

## Results and discussion

### Physicochemical properties of mineral mud

**Figure 1:**
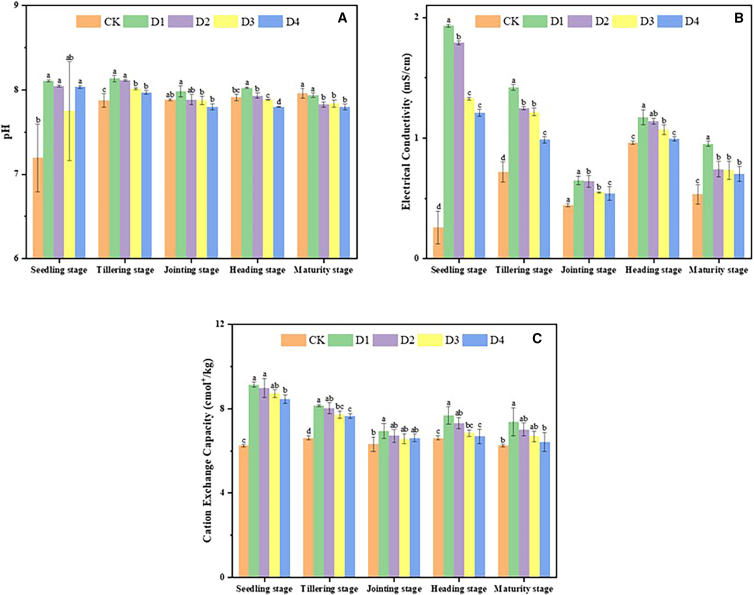
Comparison of the physicochemical properties of mineral mud at different growth stages of rice after applying amendments (A) The pH of mineral mud at different growth stages. (B) The electrical conductivity of mineral mud at different growth stages. (C) The cation exchange capacity of mineral mud at different growth stages.

**Figure 2:**
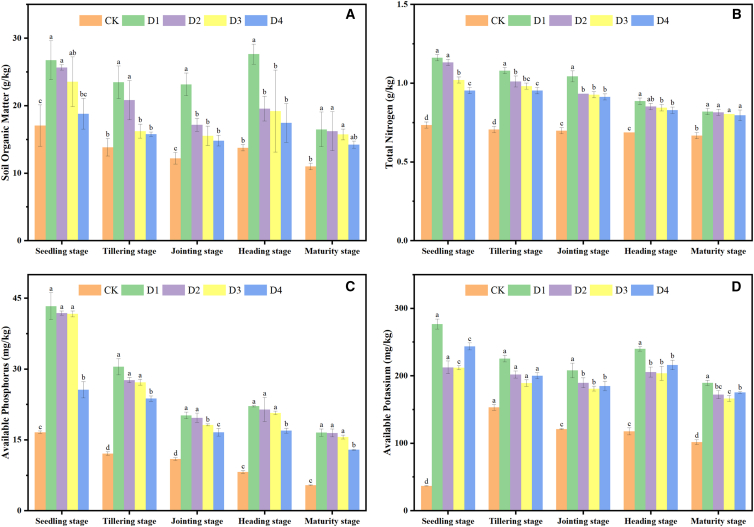
Comparison of the physicochemical properties of mineral mud at different growth stages of rice after applying amendments (A) Organic matter of mineral mud at different growth stages. (B) Total nitrogen of mineral mud at different growth stages. (C) Available phosphorus of mineral mud at different growth stages. (D) Quick-acting potassium of mineral mud at different growth stages.

Extreme pH environment directly affects the form and availability of nutrient elements, which hinders their absorption by plant roots.^26^ As shown in Figure 1A, the pH of the treated mineral mud increased to 7.87–8.13 after amendment, representing a relative increase of 9.46–13.07% compared with the unamended CK group (pH = 7.19). In a weakly alkaline environment, the solubility of Al, Cd, and Pb decreased, which reduced heavy metal migration and deposition in mineral mud, lessening the toxicity to plants.^27^ Furthermore, as the compounding ratio of HA and Si-Ca-Mg fertilizer decreased, the upward trend in pH slowed. The phenomenon is related to HA forming complexes in soil and the exchange of alkaline metal ions in Si-Ca-Mg fertilizer. The carboxyl group (–COOH) and hydroxyl group (–OH) in HA underwent coordination reactions with metal ions such as Fe^3+^ and Al^3+^ in the mineral mud to form complexes,^10^ and alkaline ions (e.g., Ca^2+^ and Mg^2+^) in the Si-Ca-Mg fertilizer underwent ion exchange with H^+^ in the mineral mud, leaving excess OH^−^. The decreasing trend in the soil pH happened during rice growth. Among the treatment groups, significant differences were observed during the seedling stage. The pH increase in D3 was notably lower than in other treatment groups. The disparity stemmed from an imbalance in the ratio between sludge and Si-Ca-Mg fertilizer. The alkalinity provided by the fertilizer was insufficient to counteract the inherent acidity of the sludge and the acidification resulting from anaerobic digestion by microbial communities, which collectively contributed to the reduced pH increase. As illustrated in Figure 1B, the amended mineral mud exhibited a significant increase in both electrical conductivity (EC) and ionic strength. As rice ripped, EC gradually decreased, which was because harmful salts in the mineral mud were leached out during drainage and irrigation, reducing the risk of mineral mud salinization.^28^ As demonstrated in Figure 1C, a significant improvement of CEC was observed in the amended mineral mud, with an increase ranging from 2.73% to 46.55%. The enhanced CEC enabled the mineral mud to retain more nutrients, adsorb, and supply essential cationic nutrients (e.g., K^+^, Ca^2+^, and Mg^2+^) for rice growth.^29^ Figure 2 shows that different amendments affected mineral mud nutrients differently. Compared with unamended mineral mud, the organic matter, available phosphorus, and quick-acting potassium in amended mineral mud increased significantly (*p* < 0.05); these nutrients reached 14.22–27.64 g/kg, 12.89–43.31 mg/kg, and 172.03–276.60 mg/kg, respectively. Total nitrogen content was 0.80–1.16 g/kg. Due to the HA top dressing during the rice jointing stage, nutrient levels rose post-joining. Per the FAO’s rice-growing guidelines,^30^ the amended mineral mud met the requirements for rice growth.

### Response of rice yield to different remediation measures

As shown in Table S1, compared with the control (CK) group, the rice yield of different treatments was significantly improved (*p* < 0.05). Among them, the combination of sludge, HA, and Si-Ca-Mg fertilizer in 3:1:3 ratio had the best growth-promoting effect on rice. Even under the adverse conditions of severe damage from birds and rodents, the yield still reached the average yield threshold of conventional farmland in Pingguo (Guangxi, China).

### Effects of different remediation measures on heavy metals in mineral mud and rice

**Figure 3:**
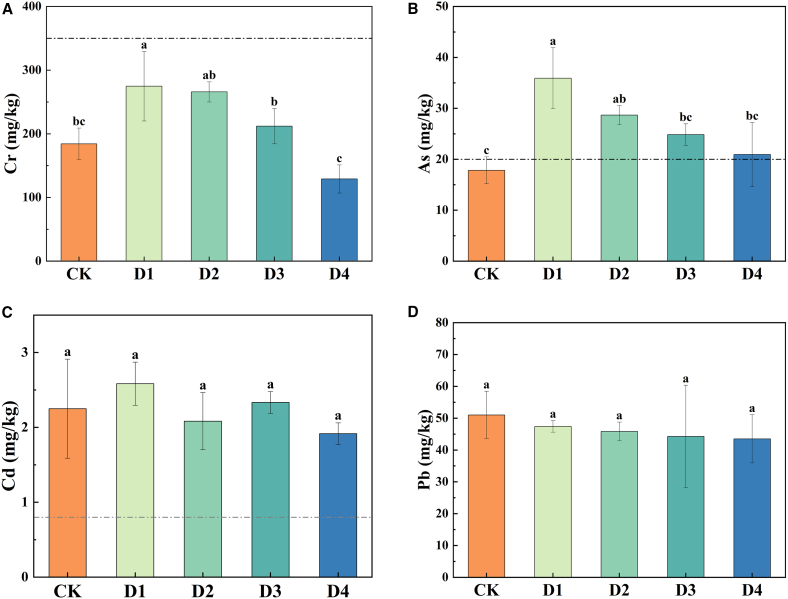
Heavy metal content in mineral mud under different amendment measures (A) Cr content in mineral mud under different amendment measures. (B) As content in mineral mud under different amendment measures. (C) Cd content in mineral mud under different amendment measures. (D) Pb content in mineral mud under different amendment measures. In (A–D), the dashed lines indicated the soil pollution risk screening values. Pb screening value is 240 mg/kg. Different lowercase letters indicate significant differences among treatments (*p* < 0.05).

In this study, soil pollution risks were assessed using GB 15618-2018,^31^ through risk screening and control values. As shown in Figure 3, Cd levels in mineral mud exceeded the risk screening value both before and after improvement but were below the control value. As levels also exceeded the screening value but not the control value after improvement. Cr and Pb levels remained below the risk screening value. Further analysis revealed that the addition of composite amendments increased Cd, Cr, and As levels, indicating that these heavy metals were present in the amendments. Dewatered sludge, part of the amendments, inherently contains various heavy metals, and its application inevitably raised the total Cd, Cr, and As contents in the mineral mud. Moreover, the heavy metal content was higher in the D1 and D2 groups than in the D3 and D4 groups, which D1–D4 denoted the experimental groups. This was mainly due to the high-background heavy metal content in the mineral mud-based paddy field conversion substrate used for ecological restoration. When amendments were added to this mineral mud, the total heavy metal content increased with the amounts of amendments applied.

**Figure 4:**
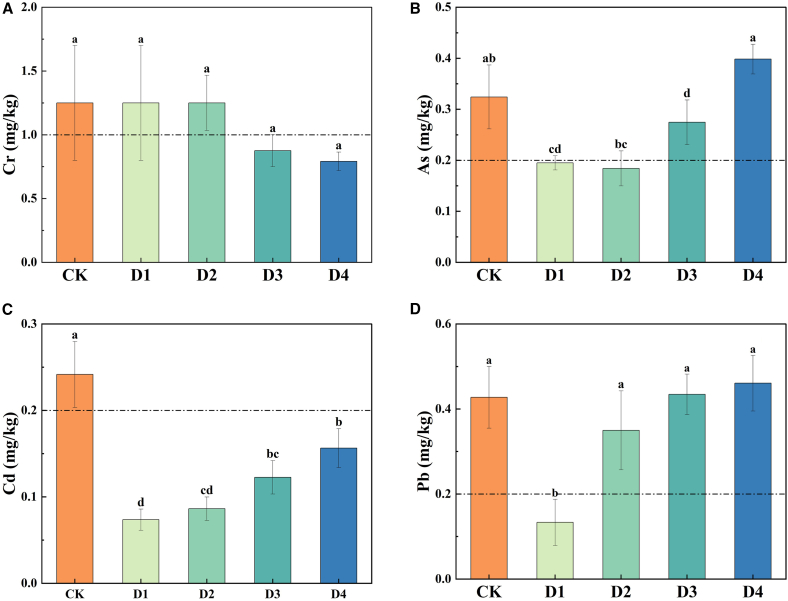
Heavy metal content in rice under different amendment measures (A) Cr content in mineral mud under different amendment measures. (B) As content in mineral mud under different amendment measures. (C) Cd content in mineral mud under different amendment measures. (D) Pb content in mineral mud under different amendment measures. In (A–D), the dotted lines indicate the maximum levels of heavy metals in rice set by the national food safety standard (GB 2762–2022), and different lowercase letters indicate significant differences among treatments (*p* < 0.05).

Figure 4 presents the average content of four heavy metals in rice under different treatments. Cd in rice showed significant differences (*p* < 0.05) among treatments, following the order CK > D4 > D3 > D2 > D1. As the application rate of the composite amendments increased, the Cd content in rice decreased. All improved treatments had Cd levels significantly below those of the CK and within the food safety limit of 0.20 mg/kg. The Cr content in rice from the CK, D1, and D2 treatments was higher than that from D3 and D4. As the amount of application of the composite amendments increased, the Cr content in rice also increased. The Cr levels in D1, D2, and CK treatments surpassed the food safety limit of 1.0 mg/kg, indicating that the composite amendments contained Cr. The Pb content in rice followed the order D4 > D3 > CK > D2 > D1, with all treatments except D1 exceeding the food safety limit of 0.20 mg/kg. The As content followed the order D4 > CK > D3 > D1 > D2, with all treatments except D1 and D2 exceeding the food safety limit of 0.20 mg/kg. Further analysis revealed that the heavy metal content in rice exceeded food safety standards due to the use of mine-washing return water for irrigation throughout the planting process. The extended planting period during the local dry season (July–October) with high temperatures, drought, and strong evaporation increased the heavy metal content in the return water. Additionally, the strong transpiration of rice worsened its heavy metal accumulation.

For the health risk assessment of heavy metals in rice, the data in Table S2 show that for both children and adults, under all improvement measures, the heavy metals Cr and As in rice have a non-cancer risk index (HQ) of greater than 1 and a cancer risk index (CR) of greater than 1 × 10^−4^. This means that there are both a non-cancer risk and a potential cancer risk. In contrast, the HQ and CR of Pb in rice under all improvement measures were less than 1 and 1 × 10^−4^, respectively, indicating that it does not cause non-cancer risks and that the cancer risk is within the acceptable range.

### The effects of different improvement measures on the richness, diversity, and composition of bacterial communities in mineral mud

After removing chimeric sequences, we conducted an in-depth analysis of sequence data at the OTU level and successfully obtained 4,428 OTUs from the five experimental fields. When the clustering threshold was set at 99%, the number of OTUs in each field varied from 697 to 2,697. Notably, the coverage ranged from 99.56% to 99.99%, indicating that over 99% of bacterial species in the samples could be detected. The addition of composite amendments significantly affected microbial abundance. As shown in Table S3, the D4 field had the highest microbial richness based on the ACE and Chao1 indices, while the CK field had the lowest. In terms of diversity, the CK field had the highest and the D3 field the lowest Shannon index, with the upward trend of Simpson index in D3, indicating that the application of the amendment facilitated the optimization of the soil microbial community.^32^

**Figure 5:**
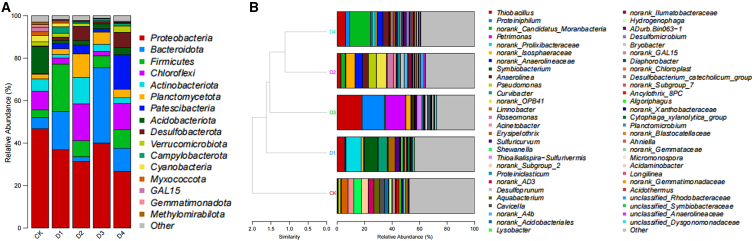
Microbial species structure at different taxonomic levels (A) Microbial species structure in different treatments at the phylum level. (B) Microbial species structure in different treatments at genus level.

As shown in Figure 5A, at the phylum level, the dominant bacterial phyla in mineral mud under different amendments were Proteobacteria (26.67%–46.78%), Bacteroidota (2.20%–35.43%), Firmicutes (3.61%–22.30%), Chloroflexi (2.13%–17.30%), and Actinobacteriota (1.67%–12.44%). As shown in Figure 5B, the unweighted pair-group method of analysis (UPGMA)-based bacterial community dendrogram clustered at 99% OTU similarity and divided the bacterial communities into four groups. Test fields D4 and D2 were in group 1, with D3, D1, and CK in separate groups. This suggests that composite amendment addition significantly altered the bacterial distribution in mineral mud. At the genus level, 55 dominant bacterial genera were identified. *Thiobacillus*, present in all fields, plays a key role in the sulfur cycle, promoting crop health and high yields. It also solubilizes insoluble phosphorus into plant-available forms, enhancing plant phosphorus nutrition.^33^ *Proteiniphilum* aids in anaerobic digestion and other biological processes, indirectly affecting mineral mud improvement.^34^ *Micromonospora* has antifungal activity and can strengthen plant immunity.^35^

## Mechanism research

### The influence mechanism of the particle size analysis and distribution in mineral mud

**Figure 6:**
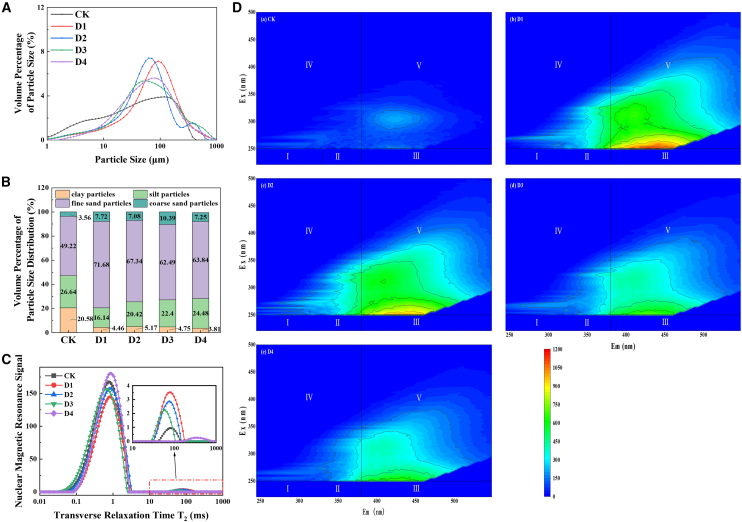
Mineral mud distribution under different amendment measures (A) Frequency curves under different amendment measures. (B) Mineral mud particle composition under different amendment measures. (C) T2 spectra under different amendment measures. (D) The regional integration diagram of DOM under different amendment measures.

The particle size frequency curve reflects the texture of mineral mud. A more uniform frequency distribution curve indicates greater particle heterogeneity.^36^ As shown in Figure 6A, both the experimental and control field mineral mud particle size frequency curves were unimodal, with particle sizes mainly concentrated between 10 and 1,000 μm. Compared with the control group, the experimental field’s frequency curve peak shifted significantly to the right, indicating a larger variation range. This suggests that the experimental field had a more uniform particle size distribution with less heterogeneity, and the soil particles were primarily concentrated between 50 and 500 μm. The improved mineral mud showed a coarsening trend in particle size, with the degree of coarsening in the order D1 > D2 > D3 > D4. The International Classification Standard was used for particle size classification in this study. Figure 6B shows significant differences in the volume percentage of mineral mud particles among different treatments (*p* < 0.05). In the control group, fine sand particles dominated at 49.22%, with coarse sand particles at 3.56%. After adding the composite amendments, the silt particle content rose to 16.14%–24.48%, and sand particles accounted for 71.09%–79.40%.

**Figure 7:**
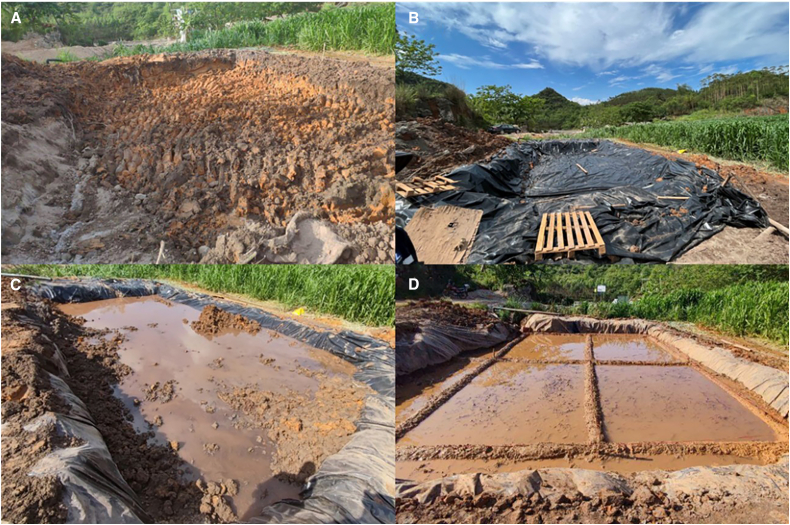
Construction of the experimental site (A) Excavation of experimental plots. (B) Laying of anti-seepage membranes. (C) Field flooding and irrigation. (D) Construction of paddy levees and drainage systems.

The amendment components promote soil particle aggregation or alter particle surface properties, making fine particles easier to cluster or be enveloped by coarse ones, thus increasing the sand-particle proportion. Meanwhile the field layout was shown in Figure 7. Among these, the D1 test field exhibited the highest percentage of sand particles. Sewage sludge, which is rich in organic matter and microbial active substances, can directly or indirectly promote the formation of soil aggregates. Organic cementation enables clay and silt particles to combine into larger aggregates. In this way, it indirectly increases the proportion of sand particles.^37^ Humic substances, through their chemical properties, use charged functional groups to provide negative charges that neutralize the positive charges on soil particles. This reduces the repulsive forces between particles and promotes the formation of micro-aggregates. Their cementation effect also stabilizes aggregates and reduces clay-particle migration and dispersion.^38^ Calcium and magnesium can replace Na^+^ or H^+^ on clay particle surfaces. This reduces clay particle dispersiveness and promotes the formation of larger aggregates.^39^ Silicate colloids can fill soil pores and combine with clay particles to form stable structures. This enhances sand particle connectivity, optimizes soil structure, and boosts soil fertility.^40^ The three components work synergistically to significantly impact soil’s physical and chemical properties, optimize soil structure, and enhance soil fertility.

### The influence mechanism of the pore distribution in mineral mud

To analyze the soil pore structure of mineral mud for its field water-holding capacity, samples from the 0–15 cm surface layer of mineral mud at maturity were saturated until reaching field water-holding capacity. The T2 spectra obtained after scanning the samples are shown in Figure 6C. The horizontal axis represents the transverse relaxation time T2. Larger T2 values indicate larger soil pore radii. The vertical axis shows the nuclear magnetic resonance (NMR) signal intensity. Stronger signals indicate more detected hydrogen protons.^41^ The peak area reflects the water content. A larger peak area means a higher water content.

The T2 spectra of mineral mud show two main peaks: a large left peak (short relaxation time) corresponding to small pores with bound water, and a smaller right peak (long relaxation time) corresponding to larger pores with free water. NMR transverse relaxation times are influenced by volume relaxation, surface relaxation, and diffusion relaxation. The pore’s specific surface area affects the relaxation time spectrum, impacting water detection in soil pores. In soil pore systems, water’s state and distribution are closely tied to pore size. In small pores, bound water interacts strongly with pore surfaces, resulting in short relaxation times. In larger pores, free water is less affected by pore surfaces and has longer relaxation times.^42^ As shown in Table S4, in the mineral mud, the CK had 81.17% bound water and 18.83% free water. Adding composite amendments gradually increased the small peak area, indicating less bound water and more free water in the experimental fields. This shows aggregate formation in the mineral mud, which improved aeration and water permeability, benefiting rice yield and quality. Studies have shown that red mud with alkaline activators can enhance binder durability, indicating that amendments can alter soil physical and chemical properties, thus influencing pore structure and water distribution.^43^

In water-based mud wells, the filtrate contacts framework minerals, and its NMR response is influenced by surface relaxation, causing signal overlap.^42^ Adding composite amendments to mineral mud may alter soil pore surface properties. This can transform bound water, influenced by surface relaxation, into free water with less surface relaxation impact. In the relaxation time spectrum, this is shown by an increase in the small-peak area. This aligns with research on water-rock interactions enhancing pore connectivity and worsening rock structure,^44^ Both indicate that added substances can change the internal structure and water status of soil or rock.

### The influence mechanism of DOM distribution in mineral mud

Dissolved organic matter (DOM) is a complex mixture of organic compounds that plays a key role in microbial metabolism and organic matter decomposition and is central to ecosystem material exchange and energy cycling. Applying organic fertilizer significantly enhances DOM’s aromaticity, humification, hydrophobicity, and molecular weight, making it more stable. Research indicates that organic fertilizer alters soil DOM composition and properties, boosts its content and stability, and stimulates microbial growth and activity, thereby influencing soil carbon cycling and greenhouse gas emissions.^45^

In Figure 6D, the three-dimensional excitation–emission matrix spectra (3D-EEMs) and fluorescence regional integration of DOM in mineral mud from different test fields are presented. Compared with the improved fields D1–D4, the CK had very weak DOM fluorescence spectra, composed mainly of two fluorescence peaks: humic-like substances (region V) and DOM metabolic products (region IV). This indicates a low DOM content in pure mineral mud. In contrast, the improved fields D1–D4 showed significantly enhanced fluorescence spectrum intensity. This demonstrates that the composite amendments increased the content of hard-to-degrade macromolecular humic-like substances in mineral mud DOM and promoted the microbial growth and activity. During microbial decomposition of organic matter and nutrient cycling, new DOM and various metabolic products are generated, enriching the DOM composition and content in mineral mud. Research shows that the DOM component in organic fertilizer can enhance soil microbial carbon utilization efficiency, promote microbial community cooperation, and alter soil nitrogen retention and availability.^46^ DOM’s fluorescence properties reflect its changing chemical composition and sources. 3D-EEMS effectively distinguishes between DOM components of different sources and characteristics, such as humic-like and protein-like substances. Organic fertilizer application introduces external DOM, increasing specific compound accumulation in soil. For example, biochar promotes the accumulation of lignin compounds with high O/C ratios and molecular stability, while straw promotes the accumulation of lignin and unsaturated hydrocarbon compounds.^45^ These changes align with enhanced DOM fluorescence spectrum intensity, indicating that composite amendments significantly impact mineral mud DOM characteristics and content.

In Table S5, the experimental fields show higher integral volumes in DOM fluorescence regions than the CK, with D1 exhibiting the most significant increase. This suggests that higher humic-like substance content in the experimental fields, especially D1, enhances nutrient availability for rice, promoting its growth. This aligns with the earlier-mentioned rice growth status.

### Conclusion

To promote the resource reutilization of mineral mud through paddy-field conversion, an organic-inorganic ternary composite amendment was developed and applied in field trials. At the optimal ratio of sludge:HA:Si-Ca-Mg fertilizer = 3:1:3, rice yields increased over 200 times relative to the original, significantly improving the growth environment. Following the application of the remediation agent, heavy metal content in rice grains remained stable or decreased significantly. Concurrently, the calculated risk indices of mineral mud declined, indicating the positive role of composite amendment in paddy field conversion. Besides, it significantly improved the originally complex remediation challenges of mineral sludge by enhancing soil structure and facilitating heavy metal migration and transformation. Although a possible pathway for the reuse of mineral mud and sludge is provided, safer utilization strategies should be further developed and investigated.

### Limitations of the study

Although this study systematically analyzed the mechanisms underlying the paddy-field transformation of mineral mud using the ternary composite amendment, certain limitations remain. First, the study examined only one growth cycle of rice, leaving gaps in the exploration of long-term changes and long-term effects. Besides, although the application of the amendment achieved an almost 200-fold increase in rice yield, the potential heavy metal risks associated with the paddy-transformed mineral mud still require further monitoring and regulation. Therefore, further optimization and exploration based on the results of this study remain promising research directions.

## Resource availability

### Lead contact

Requests for further information and resources should be directed to and will be fulfilled by the lead contact, Lihao Zhang (lhaozhang@glut.edu.cn).

### Materials availability

This study did not generate new unique reagents.

### Data and code availability

• Data: all data reported in this article will be shared by the lead contact upon request.
• Code: this study does not generate a new code.
• Additional information: Any additional information required to reanalyze the data reported in this study is available from the lead contact upon request.

## Acknowledgments

This work was supported by Guangxi Science and Technology Program (Guike LT2600640045), the 10.13039/501100012166National Key R&D Program of China (no. 2025YFC3715200), 10.13039/501100017691Guangxi Key Research and Development Program (GuikeFN2600640376), 10.13039/501100001809National Natural Science Foundation of China (52470078), Guangxi Bagui Youth Talent Support Program (GuiRenCaiBan[2024]30), and Program of Guilin Science Research and 10.13039/100006180Technology Development (20220124-1).

## Author contributions

Conceptualization and methodology, Z.Z.; investigation and writing – original draft, Z.W.; writing – review & editing, Y.K., J.L., and G.C.; funding acquisition, resources, and supervision, J.L., G.C., and L.Z.

## Declaration of interests

The authors declare no competing interests.

## STAR★Methods

### Key resources table

**Table:** 

REAGENT or RESOURCE	SOURCE	IDENTIFIER
**Software and algorithms**		

Origin	OriginLAB	V2026b, https://www.originlab.com/

**Other**		

Humic acid	Natto Biotechnology Co., Ltd., Henan, China	N/A
Si-Ca-Mg fertilizer	Sasaki Biotechnology Co., Ltd., Shandong, China	N/A
Dewatered Sludge	Yanshan Wastewater Treatment Plant, Guangxi, China	N/A
Mineral mud	Pingguo Aluminum Corporation, Guangxi, China	N/A
Malvern laser particle size analyzer	Malvern Panalytical, UK	Mastersizer 3000
Low-Field Nuclear Magnetic Resonance	Shanghai Electronic Technology Co., LTD, China	MesoMR23-060H-I
Three-dimensional fluorescence spectrometer	HORIBA Scientific, Japan	Aqualog-UV-800

### Method details

#### Experimental design

The field trial was conducted at Pit No. 18 (23°19′ N, 107°35′ E) of the Pingguo Aluminum Corporation (Guangxi, China). Mining activities at this site ceased in 2006, with the pit covering an area of approximately 7,000 m^2^. In November 2022, the pit was backfilled with mineral mud, which underwent consolidation and drainage treatment prior to the experimental application. The digital photos of the field layout for the field experiment shown in Figure 7, and details of the mineral mud within the field presented in Table S6.

As illustrated in Figure S1, a rectangular experimental field (11 m × 7.5 m) was built using a randomized block design and divided into four standard plots (5 m × 3.3 m) and one control plot. The 0.3-meter-wide ridges between plots were covered with high-density polyethylene film to prevent nutrient migration and had independent drainage outlets for precise water control. Each plot was filled with the mixture of 5.2 t of mineral mud (Based on the volumetric weight and water content of the mineral mud, the weight was calculated corresponding to a backfilling depth of 20 cm) and certain amounts of ternary composite amendments. The composite amendment was formulated from sludge, humic acid (HA), and Si-Ca-Mg fertilizer (SCM) according to the ratios shown in Table S7. The silicon-calcium-magnesium fertilizer was used to adjust soil pH, improve soil physicochemical properties, and simultaneously enhance soil cation exchange capacity while forming new complexes to mitigate heavy metal toxicity. The sludge used was collected from the Yanshan Wastewater Treatment Plant in Guangxi, China. In order to eliminate the influence of pathogens, it underwent routine dewatering and anaerobic digestion treatment at the wastewater treatment plant. The physical properties and composition of the calcium-magnesium fertilizer and sludge are provided in the supporting information. Humic acid serves as an activator, promoting plant nutrient absorption while optimizing soil structure to make it suitable for plant growth. Furthermore, humic acid’s structure can immobilize heavy metals in soil, reducing crop uptake of these metals and minimizing human health risks. During the experiment, 50% of HA was incorporated as a base fertilizer through homogeneous mixing with mineral mud, while the remaining 50% was applied as a topdressing fertilizer during the rice jointing stage.

#### Mineral mud sampling and processing

The sampling procedure was conducted using the five-point sampling method during the rice seedling, tillering, jointing, heading, and maturity stages. Soil samples from 0 to 20 cm depth were collected and transported to the laboratory in an insulated container with ice packs. A portion of the mineral mud was stored under −80°C, while the remainder was air-dried, sieved, homogenized, and cleared of plant residues, roots, stems, and stones for subsequent physicochemical analysis. The sampling depth was set to encompass the primary zone of activity for the vast majority of rice root systems.^47^

#### Analytical methods

The particle size distribution of mineral mud was determined using Malvern laser particle size analyzer. The pore distribution of mineral mud was measured with a low-field nuclear magnetic resonance instrument. Absorption and three-dimensional fluorescence spectrometer scanning spectrophotometers were used to measure the DOM in mineral mud. The distribution of DOM was analyzed by calculating the integral volume and standard integral volume of each fluorescent region in the three-dimensional fluorescence spectra using the fluorescence regional integration method.

#### Health risk assessment

The health risk assessment employed the model proposed by the U.S. EPA.^48^ This model is used to evaluate the non-carcinogenic and carcinogenic risks for children and adults resulting from the consumption of rice. The calculation formula is as follows:

(Equation 1) $$\text{ADI}=\frac{C\times \text{IR}\times \text{EF}\times \text{ED}}{\text{BW}\times \text{AT}}$$

(Equation 2) $$\text{HQ}=\frac{\text{ADI}}{\text{RfD}}$$

(Equation 3) CR=ADI×SF

Where, ADI was the average daily intake of heavy metals via rice consumption (mg/(kg·d)).

C was the concentration of the contaminant in rice (mg/kg).

IR was the daily intake of rice by the population in the study area (kg/d).

EF was the annual average exposure frequency of the population (d/a).

ED was the exposure duration of the population (years).

BW was the average body weight of the population in the study area (kg).

AT was the average lifetime of the population (d).

RfD was the reference dose for heavy metal exposure.

SF was the cancer slope factor. Detailed index value was shown in Table S8.

HQ was the hazard quotient for a single heavy metal, representing non-carcinogenic risk:

HQ < 1 indicated no significant non-carcinogenic risk.

HQ > 1 indicated the presence of such risk.

CR was the cancer risk index for a single heavy metal:

CR below 10^−6^ indicated no carcinogenic risk.

CR above 10^−4^ indicated an unacceptable carcinogenic risk for humans.

The value between above two thresholds indicated an acceptable carcinogenic risk.

### Quantification and statistical analysis

Error analysis and significance analysis in the study were both performed using Origin 2026b and Paired Comparison Plot (built-in plugin). Error bars in Figures 1, 2, 3, and 4 were expressed as mean ± SD for error analysis. Significance analysis was based on the Dunn-Šidák method for mean comparison, letter markers in Figures 1, 2, 3, and 4 and Table S2 indicated *p* < 0.05.
