# Supplementary material for: Ternary composite amendments of sludge-humic acid-fertilizer for mineral mud paddy field utilization
Source: iScience. 2026 Jul 3;29(7):116551. doi: 10.1016/j.isci.2026.116551 (PMC13378371; doi:10.1016/j.isci.2026.116551)
Supplement: Document S1. Figure S1 and Tables S1–S8 [file mmc1.pdf]

iScience, Volume *29*

Supplemental information

Ternary composite amendments of sludge-humic acid-fertilizer for mineral mud paddy field utilization

Zongwei Zhu, Zhihao Wang, Yusong Kong, Jing Liang, Gongning Chen, and Lihao Zhang

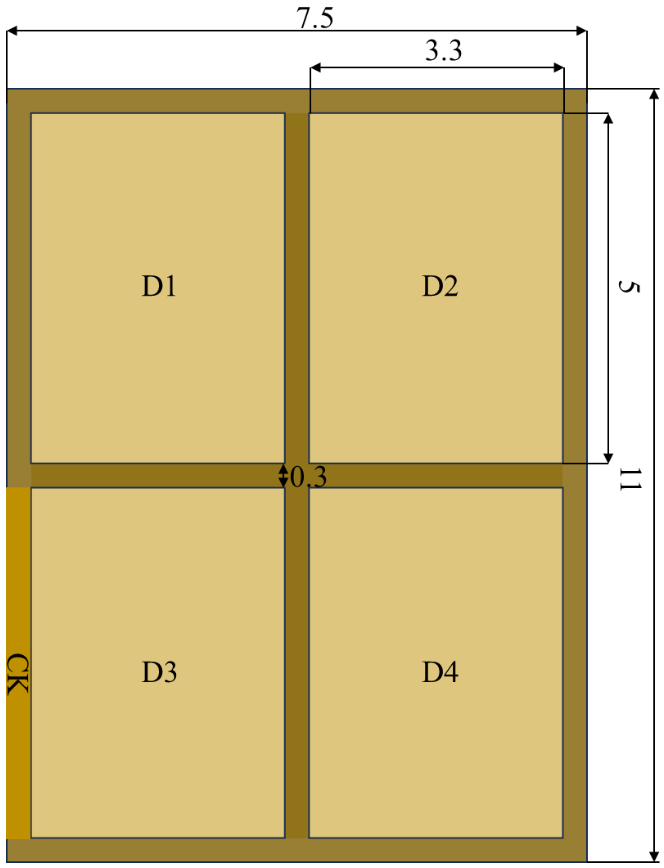

Figure. S1 Aerial view of the experimental site. Related to *Experimental Design*.

Table S1. Rice yield and yield components across experimental treatments. Related to Response of rice yield to different remediation measures.

Group	Plant Height (cm)	DW_A (g)	DW_B (g)	Tillers	Yield (kg/hm^2)
CK	53.3d	4.4c	0.5b	4.3c	20.87e
D1	134.9a	85.1a	14.1a	14.3a	4780.01a
D2	130.6ab	58.8b	13.0a	12.3a	3880.09b
D3	122.6bc	55.1b	8.6ab	10.3ab	2960.45c
D4	115.2c	46.0b	8.0ab	7.3bc	1800.28d

Group	Effective panicles	Grains Per Panicle	Thousand-Grain Weight (g)	Grain set ratio (%)
CK	15.3c	55.0d	16.10b	21.60b
D1	77.7a	257.0a	25.89a	93.31a
D2	76.0a	231.7b	24.80a	88.88a
D3	61.3ab	229.7bc	24.17a	87.56a
D4	51.0bc	213.0c	18.73ab	88.63a

Where, DW_A was above-ground dry weight, DW_B was below-ground dry weight. Different lowercase letters indicate significant differences among treatments ($P < 0.05$)

Table S2. Health risks of heavy metal intake in rice. Related to Figure 3 and Figure 4.

Index	Object	Element	CK	D1	D2	D3	D4
HQ	Children	Cd	1.76E+00	5.37E-01	6.29E-01	8.94E-01	1.14E+00
		Cr	3.04E+00	3.04E+00	3.04E+00	2.13E+00	1.92E+00
		Pb	8.66E-01	2.70E-01	7.08E-01	8.80E-01	9.33E-01
		As	7.88E+00	4.74E+00	4.47E+00	6.67E+00	9.68E+00
	Adults	Cd	1.09E-03	3.32E-04	3.90E-04	5.54E-04	7.06E-04
		Cr	1.88E+00	1.88E+00	1.88E+00	1.32E+00	1.19E+00
		Pb	5.36E-01	1.67E-01	4.39E-01	5.45E-01	5.78E-01
		As	4.88E+00	2.93E+00	2.77E+00	4.13E+00	6.00E+00
CR	Children	Cd	1.07E-02	3.27E-03	3.84E-03	5.45E-03	6.96E-03
		Cr	9.11E-03	9.11E-03	9.11E-03	6.38E-03	5.77E-03
		Pb	2.65E-05	8.26E-06	2.17E-05	2.69E-05	2.86E-05
		As	3.54E-03	2.13E-03	2.01E-03	3.00E-03	4.36E-03
	Adults	Cd	6.66E-03	2.03E-03	2.38E-03	3.38E-03	4.31E-03
		Cr	5.65E-03	5.65E-03	5.65E-03	3.95E-03	3.58E-03
		Pb	1.64E-05	5.12E-06	1.34E-05	1.67E-05	1.77E-05
		As	2.20E-03	1.32E-03	1.25E-03	1.86E-03	2.70E-03

Table S3. Microbial community richness and diversity of alpha-index in different samples. Related to Figure 5.

Group	Coverage	ACE	Chao 1	Shannon	Simpson
CK	0.9999	699.94	702.00	5.5588	0.0107
D1	0.9994	1846.23	1840.39	5.1251	0.0266
D2	0.9979	1970.83	1932.02	5.2257	0.0170
D3	0.9968	2133.70	2046.84	4.1835	0.0711
D4	0.9956	2833.25	2744.72	5.4480	0.0292

Table S4. Peak area distribution of mine mud characteristic peaks under different amendment. Related to Figure 6.

Group	Large peak area	Small peak area	Percentage of large peak area (%) (Bound water)	Percentage of small peak area (%) (Free water)
CK	260.04	60.33	81.17	18.83
D1	231.60	315.64	42.32	57.68
D2	279.54	206.34	57.53	42.47
D3	215.99	108.56	66.55	33.45
D4	289.14	98.60	74.57	25.43

Table S5. Fluorescence regional integral volume of mine mud dom under different amendment measures (Φ_i) × 10^6/au·nm^2. Related to Figure 6.

Region / Group	Dissolved Organic Matter Metabolites (Region IV)	Humic-like Substances (Region V)
CK	0.43	1.30
D1	1.83	10.08
D2	1.82	8.89
D3	1.24	5.92
D4	1.11	5.24

Table S6. Basic physicochemical properties of mineral mud. Related to *Experimental Design*.

Index	Value	Index	Value
Volumetric Weight (g/cm^3)	1.12 ± 0.021	Cation Exchange Capacity ($cmol^+/kg$)	7.79 ± 0.039
Specific Gravity (g/cm^3)	2.75 ± 0.051	Organic Matter (g/kg)	7.5 ± 0.100
Porosity (%)	54.09 ± 0.080	Total Nitrogen (g/kg)	1.33 ± 0.115
pH	6.93 ± 0.012	Available Phosphorus (mg/kg)	3.1 ± 0.180
Conductivity (mS/cm)	0.15 ± 0.002	Available Potassium (mg/kg)	24 ± 1.029

Table S7. Gradient design of amendment ratio. Related to *Experimental Design*.

Experimental group	Sludge (g/kg)	Humic Acid (g/kg)	Fertilizer (g/kg)
CK	-	-	-
D1	30	10	30
D2	30	7.5	20
D3	30	5	10
D4	20	10	20

Table S8 Parameters of risk exposure model Related to *Health risk assessment*.

Index	Heavy metal	Child	Adult
IR	-	0.14	0.28
ED	-	10	30
EF	-	365	365
BE	-	19.2	62
AT	-	10 × 365	30 × 365
RfD	Cd	0.001	-
	Cr	0.003	-
	Pb	0.0036	-
	As	0.0003	-
SF	Cd	-	6.1
	Pb	-	0.0085
	As	-	1.50
